# Exploring Combinations of Auditory and Visual Stimuli for Gaze-Independent Brain-Computer Interfaces

**DOI:** 10.1371/journal.pone.0111070

**Published:** 2014-10-28

**Authors:** Xingwei An, Johannes Höhne, Dong Ming, Benjamin Blankertz

**Affiliations:** 1 Department of Biomedical Engineering, Tianjin University, Tianjin, China; 2 Neurotechnology Group, Berlin Institute of Technology, Berlin, Germany; 3 Machine Learning Group, Berlin Institute of Technology, Berlin, Germany; CEA.DSV.I2BM.NeuroSpin, France

## Abstract

For Brain-Computer Interface (BCI) systems that are designed for users with severe impairments of the oculomotor system, an appropriate mode of presenting stimuli to the user is crucial. To investigate whether multi-sensory integration can be exploited in the gaze-independent event-related potentials (ERP) speller and to enhance BCI performance, we designed a visual-auditory speller. We investigate the possibility to enhance stimulus presentation by combining visual and auditory stimuli within gaze-independent spellers. In this study with N = 15 healthy users, two different ways of combining the two sensory modalities are proposed: simultaneous redundant streams (Combined-Speller) and interleaved independent streams (Parallel-Speller). Unimodal stimuli were applied as control conditions. The workload, ERP components, classification accuracy and resulting spelling speed were analyzed for each condition. The Combined-speller showed a lower workload than uni-modal paradigms, without the sacrifice of spelling performance. Besides, shorter latencies, lower amplitudes, as well as a shift of the temporal and spatial distribution of discriminative information were observed for Combined-speller. These results are important and are inspirations for future studies to search the reason for these differences. For the more innovative and demanding Parallel-Speller, where the auditory and visual domains are independent from each other, a proof of concept was obtained: fifteen users could spell online with a mean accuracy of 87.7% (chance level <3%) showing a competitive average speed of 1.65 symbols per minute. The fact that it requires only one selection period per symbol makes it a good candidate for a fast communication channel. It brings a new insight into the true multisensory stimuli paradigms. Novel approaches for combining two sensory modalities were designed here, which are valuable for the development of ERP-based BCI paradigms.

## Introduction

Brain-computer interfaces (BCIs) can provide direct communication by non-muscular methods for people with severe motor impairments [1], [2]. Most BCI systems either are based on modulations of local brain oscillations (mostly sensorimotor rhythms (SMRs)) that are induced by certain voluntary control strategies such as Motor Imagery based techniques [3], [4], or they exploit event-related potentials (ERPs) that are modulated according to the allocation of attention to selected stimuli. While SMR-based BCIs have the advantage of providing a continuous control signal (in time and magnitude), ERP-based BCIs are commonly considered to be more stable [5] and more efficient for selection tasks, such as mental typewriting. In ERP-based spellers, users can select symbols by directing their attention to stimuli, from the visual, auditory or tactile domain.

The Matrix Speller designed by Farwell and Donchin [6] was the first approach to provide communication to users with severe motor disabilities based on ERPs. It is remarkable that this early approach is still popular, and many novel variants have been devised that still follow the original idea quite closely. Some of these approaches have optimized the exploitation of visual evoked potentials (VEPs) that are elicited by stimuli within the foveal field [7], [8]. Also, it is the Matrix Speller that is employed in one of the rare cases of 'home use' BCIs by a paralyzed user [9]. However, it was shown in [10], [11] that the performance of the Matrix Speller depends critically on the user's ability to fixate the target character which limits its applicability to users with a certain degree of oculomotor control. To also accommodate users with impaired ocular motor control, recent studies proposed alternative paradigms to implement gaze-independent visual BCI spellers, see [12], [13], [14], [15], [16]. For an overview of gaze-independent spellers see [17].

As an alternative paradigm for users with limited or even no vision several research groups investigate spellers based on tactile or somatosensory [18], [19] and auditory [20], [21], [22] stimuli. Paradigms with somatosensory stimuli were considered as a suitable alternative for vision and/or hearing impaired BCI users. Studies about auditory stimuli [20], [21], [22] examined approaches similar to the visual matrix speller, mapping different sounds to rows and/or columns of the symbol matrix, such as the words from 1 to 10 (for a 5*5 matrix), and 6 environmental sounds (for 6*6 matrix). A novel approach that allowed considerably higher transmission rates was proposed by Schreuder and colleagues [23], [24]. The key idea was to employ spatially distributed auditory stimuli, which allowed a fast presentation speed and an easier allocation of attention. Höhne et al. [25], [26] introduced a variant of that approach that uses less spatial directions but adds pitch or just the sound of the letters as independent features. More recently, it was shown that the use of syllables as natural stimuli not only improved the users' ergonomic ratings but also increased the classification accuracy [27]. Klobassa et al. [21] used a multimodal audio-visual speller paradigm to provide a better 'training' in initial sessions to finally use mono-auditory speller.

Instead of uni-modal stimuli paradigms, researchers started to focus on using bimodal stimuli [18], [28], [29], [30], [31]. Talsma and colleagues [32] reviewed the developments in the understanding of the interaction between attention and multi-sensory processing, focusing on studies using audio-visual stimulus material. Their review also identified several important directions and challenges for future research in this field. Teder-Sälejärvi et al. [28] used randomized sequences of unimodal (auditory (**A**) or visual (**V**)) and simultaneous bimodal (**AV**) stimuli presented to right- or left-field locations. The results in that study showed overlapping but distinctive patterns of multisensory integration for spatially congruent and incongruent **AV** stimuli. Belitski and his colleagues [30] presented an extension of the matrix speller using a so-called 'visual+auditory' paradigm as a transient process for best performance and moved smoothly to purely auditory. Results demonstrated the effectiveness of this approach with this transient process. It was also found that the ‘visual + auditory’ stimuli increased the average strength of the stimulus response in matrix-speller style BCIs, when compared to unimodal stimuli. However, this study refers only to gaze-dependent visual spellers. Though it was not designed for ERP spelling, Thurlings and her team [31] investigated the effect of bimodal visual-tactile stimulus presentation on the ERP components, BCI performance and participants' task performance. Results of this study showed enhanced early components (N1), which may enhance the BCI performance, while also showed reduced late ERP components (P300).

We live in a multisensory world in which we are continuously deluged with stimulus input through multiple sensory pathways [32]. Therefore it is important to address the following questions: (1) Can multi-sensory integration be exploited in a gaze-independent ERP speller in order to enhance BCI performance? (2) Is there any difference of brain response between single and multi-sensory stimuli? (3) Is it possible to use a BCI paradigm with two independent channels, coding different information?

To answer these questions, we designed a visual-auditory speller (called Combined-Speller, denoted as **AV**) by simultaneous presentation of visual stimuli and auditory stimuli. To enable a comparison with the uni-modal speller paradigms, the mono-visual (denoted as **V**) and mono-auditory (denoted as **A**) spellers are also studied in addition. For answering the third question, we propose a new and truly multi-modal BCI approach (called Parallel-Speller, denoted as V*A). In the Parallel-Speller, the visual and auditory stimuli are coded independently.

## Material and Methods

### Participants

Fifteen healthy subjects (8 female) aged 24-34 (mean 26.9±2.63 years) participated in this study. Two of the participants had already participated in earlier BCI experiments. Each participant did not suffer from a neurological disease and had normal hearing. They also provided written informed consent confirming the notification of the experimental process, the using of the data and the personal right of themselves. Subjects were paid for their participation with 8€/hour, and the entire experiment lasted 3 to 4 hours. The study was approved by the Ethics Committee of the Charité University Hospital (number EA4/110/09).

### Stimuli

**Figure 1:**
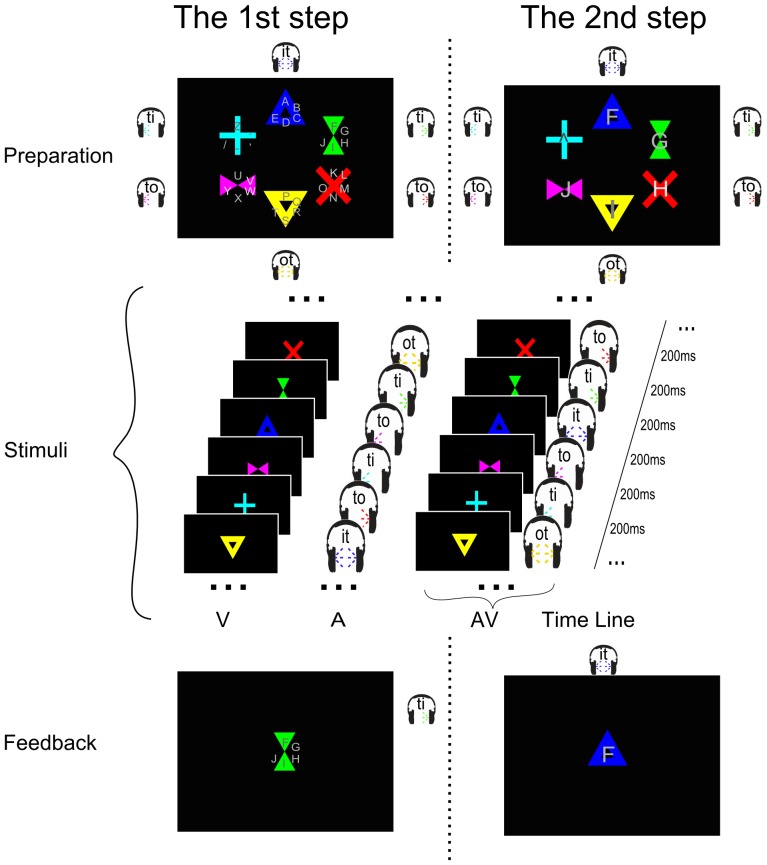
Visualization of the experimental design of the experiment for unimodal speller (Visual: V and Auditory: A) and Combined-Speller (AV). The left column shows the first selection period for group selection. The right column shows the second selection period for symbol selection. The upper channel shows the ‘target cue’ part before stimuli start, including the cue for showing the position (or voice) of the target and the countdown part for hint of the start. The middle panel shows the time sequence of each stimuli paradigm. The lower panel shows the feedback after each selection period. For the first selection period, the group of the target symbol was chosen, and the symbols in that group will re-distributed into these six visual shapes (the last shape, light blue cross, was left blank as the ‘backdoor’ symbol) according to their position in the chosen group.

**Figure 2:**
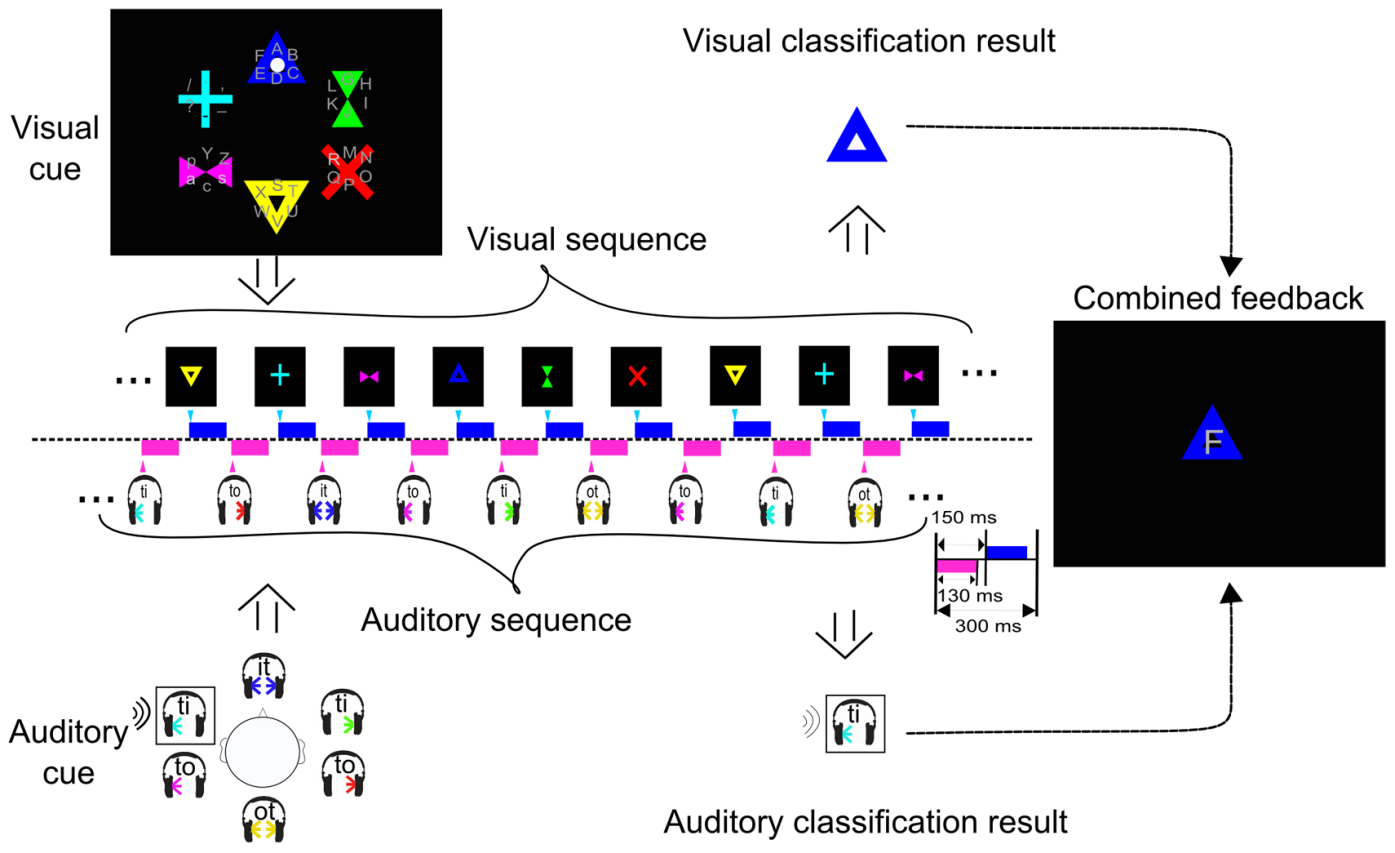
Visualization of the experimental design of the experiment for Parallel-Speller (V*A). Six symbols locate in each group, making a total of thirty-six symbols. The presentation of the visual stimuli was used to choose the group the symbol was in. The position of the symbol in the group was selected through the auditory domain. The upper panel shows the sequence of visual target selection, and the lower panel shows that of the auditory target selection.

This study compares four different conditions related to sensory modalities that can be used to drive a BCI speller. Two conditions use stimuli in one sensory modality only, labeled **V** for visual speller and **A** for auditory speller. These experimental settings are similar to existing speller paradigms and have been used for comparison with the novel multimodal spellers. The speller of the **AV** condition (Combined-Speller) uses simultaneous auditory and visual stimuli as redundant information, while the Parallel-Speller (V*A condition) exploits alternating auditory and visual cues as independent streams. Figure 1 and Figure 2 show the course of the experiment. The three conditions **V**, **A**, and **AV** used a vocabulary of thirty symbols and a two periods selection procedure as explained below (Figure 1), while condition V*A allows to select one out of thirty six symbols within a single selection period (Figure 2). The thirty-symbol alphabet comprised the standard Latin alphabet, punctuation marks '.' and ',', a space symbol '_' and a backspace symbol '<' that could be used to erase the previous symbol. The thirty six-symbol alphabet extended the thirty-symbol alphabet by additional control keys (lower cases ‘s’ for ‘Shift’, ‘c’ for ‘Ctrl’, ‘A’ for ‘Alt’, ‘p’ for ‘Space’) and punctuation marks (‘-’ and ‘?’).

In all conditions, the selection of one symbol is coded by two selection steps of one out of six targets each. In the first selection step, a group of symbols (e.g., 'ABCDE') is selected, while in the second selection step one symbol of that group is selected. In the condition V*A both steps are performed at the same time (one by each modality), while in the other speller they are performed subsequently. For the serial selection, each group contains five characters (or symbols) and one symbol (‘∧’) that serves as a backdoor to return to the group selection step that can be used in case of an erroneous group selection. In the concurrent selection of group and symbol in condition A*V, the backdoor functionality makes no sense. Therefore, additional symbols were used as replacement. (A different option would have been to have only five symbols per group such that the symbol stage would only be a five class selection. This would have been an advantage with respect to the accuracy of the Parallel-Speller. However, as the focus of this study was the comparison of the multimodal speller with the unimodal spellers, the preference was given to keep the complexity of the selections constant.).

It is important to note that before each selection period, a countdown was launched. It included the intensification of the visual and (or) auditory target stimuli for three times. This was followed by the pre-flashing of the last 3 digits (3, 2, 1) synchronized with the subsequent stimulus sequence. The design of the countdown part did not only provide a cue for the targets but also helped the participant to get used to the flashing frequency.

In condition **V**, visual stimuli were presented as in the Center Speller [12]. The Center Speller is a gaze-independent speller, where participants attend to the center of the screen at which the 6 stimuli are presented in a pseudo-random sequence. Six different stimuli were designed such that they had a unique geometrical shape and color (Figure 1), providing two distinct features for visual discrimination. During the presentation of the stimuli, the geometrical shapes were presented centrally in a sequential fashion. The duration of each stimulus was 130 ms and the stimulus onset asynchrony (SOA) was kept at 200 ms. The reason for choosing 130 ms as the duration of the visual stimuli was to match the duration of the auditory stimuli (see below).

In condition **A**, auditory stimuli were presented in a similar fashion as in [25]. We used only 6 stimuli in this study (Figure 1). Short spoken syllables (left 'ti', left 'to', middle 'it', middle 'ot', right 'ti', right 'to') were sung with different pitches by three speakers (bass, tenor and soprano human voices) as stimuli. Every speaker was presented only from one fixed direction (bass: from the left, tenor: from the middle, soprano: from the right). For each direction we recorded 2 stimuli (stimuli with vowel ‘i’ and stimuli with vowel ‘o’). The stimuli with the vowel 'i' (left ‘ti’, middle ‘it’ and right ‘ti’) were recorded with high pitch (A#) and the stimuli with vowel 'o' (left ‘to’, middle ‘ot’ and right ‘to’) were recorded with comparatively low pitch (C#). All of the auditory stimuli were kept within 130 ms as the duration of the visual stimuli. The SOA was also chosen to be 200 ms, such that conditions **V** and **A** were the analog in most aspects but used different sensory modalities.

In condition **AV**, corresponding visual and auditory stimuli were presented simultaneously, while those two concurrent streams coded the same information, see Figure 1. Therefore, targets in the visual sequence always appear at the very same time as targets in the auditory sequence. Senkowski et al. reported the timing of multimodal stimuli to be a crucial aspect [33]. Thus, we investigated the optimal delay in a pilot experiment and finally set the timing that auditory stimuli were presented about 17 ms (1 fame of a 60 Hz monitor) earlier than the corresponding visual stimuli. Participants were instructed to concentrate on the combined visual and auditory stimuli at the same time.

During conditions **V**, **A**, and **AV**, the order of stimuli was randomized within each repetition with the constraint that the same stimulus could not appear with less than two different stimuli in between. Participants were instructed to count the number of target occurrences to help them focus on the stimuli.

In condition V*A, the same visual and auditory stimuli have been used, but they were presented as independent sequences. The SOA in each sequence was 300 ms and the onset between auditory and visual cues was 150 ms, with 130 ms duration of each stimuli, see Figure 2. To spell a letter, the group was selected through visual stream, while the within-group selection was done via the auditory domain. That way, two independent decisions are made parallel. For example, to select the symbol 'F', which is the sixth symbol in the first group (cf. Figure 2 visual cue), the target in the visual sequence is the blue triangle and in the auditory sequence the base 'ti' (cf. Figure 2 auditory cue). As it was described above selections of visual and auditory targets were made simultaneously and independently. However those two channels stimuli were alternatively presented in a rapid sequence (see Figure 2 visual and auditory sequences). After several presentations of each stimulus (10 repetitions were used in this experiment), the binary classification and multiclass selection was performed independently for each stimulus domain – resulting in a visual and an auditory output. Combining the visual and auditory output, the final symbol was spelled (see Figure 2).

Note that due to the independence of the two streams, targets in the visual and in the auditory sequence occur at different times in both sequences. The minimum inter-target distance within each modality (visual target to visual target or auditory target to auditory target, cf. Figure 2) is three stimuli and equivalently 900 ms. However, it may happen that two target presentations are only 150 ms apart (e.g. the time between a visual target and auditory target). This cannot be circumvented, as any combination of group and symbol-with-group can be the pair of targets.

### Procedure

Visual stimuli were presented on a 19' TFT screen with a refresh rate of 60 Hz and a resolution of 1280*1024 px^2.^ Auditory stimuli were presented through a light neckband headphone (Sennheiser PMX 200) that was positioned comfortably. Participants were seated in a comfortable chair at a distance of about 1 m from the screen. During the preparation of the EEG, the written and verbal instructions were provided. Thereafter, subjects were instructed to sit still, relax and try to minimize eye movements (try not to blink, though it is inevitable) during the course of a trial.

There were 2 offline calibration runs for each the conditions **V**, **A**, and **AV**. In each calibration run, participants are provided with a sequence of 6 symbols for which they have to perform the 'selections' by allocating attention to the corresponding target stimuli. In total, there were 12 symbols (6 symbols * 2 calibration runs) for each condition in the calibration phase. The sequence of symbols to spell was randomly selected from all available symbols, and this differed between subjects. The 6 runs of the 3 conditions (**V**, **A**, **AV**) were conducted in pseudo-randomly order.

After the calibration phase, offline analysis was conducted to train a classifier (see [34] and Section 2.4) on the collected data of condition **AV**. After a short pause, participants were instructed to complete one self-conceived word (without telling the experimenter) in free spelling mode comprising 6–10 symbols with condition **AV**. During free spelling, participants could use the backdoor symbol '∧' (which is contained in every group) to cancel the selection of the group and use the backspace symbol ('<') to erase the previous symbol, if a wrong group or symbol was selected. Free-spelling was conducted as motivation and for getting familiar with online spelling. Thereafter, participants were asked to spell twenty predefined symbols in the so-called copy-spelling mode (again condition **AV**). In contrast to free-spelling, erroneously selected symbols needed not to be deleted as in the free spelling part. As a two-period spelling paradigm, the spelling in condition **AV** faces the problem that the first period (group selection) could be wrong, which will definitely lead to wrong symbol selection. In this study, the default visual and auditory targets in the second period were the same as the correct target should be, no matter the correct group was selected or not. From the symbols the participants selected, it is easy to analyze the accuracy in each period.

Condition V*A was conducted after a short break. The reason for having this condition always at the end was that participants should have experienced the simpler conditions first in order to be able to cope with this more demanding condition. This training effect was conjectured to outweigh the possible detrimental effect of fatigue that may occur at the end of the experiment. Participants underwent a calibration of 4 runs (6 symbols in each run), a free spelling phase where self-chosen words could be spelled, and finally the copy spelling of a predefined sentence.

In the calibration phase, there were similar countdown and feedback parts for each trial to select the target symbol. The intensification of the target in the countdown part was a flashing circle in the center of the target visual shape with the target auditory stimuli presented through the earphone (both for three times), followed by the pre-flashing of the last 3 digits (3, 2, 1) synchronized with the subsequent stimulus sequence. Only one selection period was needed to choose the target symbol. The visual stimuli and auditory stimuli were presented independently and alternatively as described in Section 2.2. Considering the complexity of the task, it is difficult to quickly count the number of targets occurrences. There were 20 target occurrences in 18 s. It is possible that visual target and auditory target may occur consequently. In this circumstance, participants need to count two numbers in 600 ms. Thus participants were not asked to count the number target occurrences, but just to pay attention to the target stimuli in the auditory as well as in the visual stream. After the presentation of 10 repetitions of the stimuli (since the task is difficult than other paradigms, more repetitions were used here), the outputs of the visual and auditory classification were made according to these two stimuli streams.

The offline analysis for condition V*A were conducted after the calibration phase, followed by free spelling and copy spelling runs. During free spelling, the participants could spell any word without telling the experimenter, and could use the backspace symbol ('/') to erase the previous symbol, if a wrong symbol was selected. Thereafter, twenty predefined symbols (the same as in condition **AV**) in the so-called copy-spelling mode without erasing the wrong symbols selected.

For this whole experiment, we have ten calibration runs in total. Each calibration run lasts for less than 3 minutes, while 6 symbols are spelled. For conditions **V**, **A** and **AV**, there are 2 selection periods for choosing a symbol, compared to only one selection period for per symbol in condition V*A. Besides the calibration runs, for condition **AV** and V*A, free spelling and copy spelling runs were also conducted after their calibration runs. Neither copy spelling nor free spelling was done with unimodal stimuli (condition **V** and **A**). Between each run, participants could rest for 2–5 min. There is no break within runs.

### Data acquisition and analysis

Electroencephalogram (EEG) signals were acquired using a Fast'n Easy Cap (EasyCap GmbH, Munich, Germany) with 63 Ag/AgCl electrodes placed at the standard positions of the international 10–20 system. Channels were referenced to the nose, with the Ground electrode located at the front (at position AFz). Electrooculogram (EOG) signals were recorded additionally. Signals were amplified using two 32-channel amplifiers (Brain Amp by Brain Products, Munich, Germany), sampled at 1 kHz. Further online and offline analysis was performed in Matlab. Statistical analysis was performed with both IBM SPSS statistics 20 and MATLAB. The visual and auditory feedback was implemented using the open source framework PyFF (Venthur et al., 2010).

To evaluate the workload of each condition, participants were asked to fill in the NASA TLX questionnaire (NASA Human Performance Research Group, 1987) after the calibration phase of each condition. It was introduced as a measure of usability in BCI by Pasqualotto et al. [35] and Riccio et al. [36], and it has been used to compare the workload for a visual and an auditory speller by Käthner et al. [37]. It was used here as a multidimensional rating procedure to derive an overall workload score based on a weighted average of ratings on six sub-scales (Mental workload, Physical workload, Temporal workload, Performance, Effort needed and Frustration) for each condition. There were fifteen pair-wise comparisons of each two of the six subscales (Mental vs. Physical, Mental vs. Temporal, and Physical vs. Temporal, et al.) for the participants to choose the subscale in the comparison which weighs more for the whole workload. The subscale wins in each pair-wise comparison will count 1. So the sum score of the weightings is fifteen. A high score reveals an increased importance for workload.

Generally, EEG is prone to various sources of noise arising from factors such as 50 Hz power noise or ECG artifacts. Moreover, the discriminative components of the ERP are mainly found below 40 Hz. A Chebychev filter was therefore applied for offline analysis, using a passband up to 40 Hz and a stopband starting at 49 Hz and then down sampled to 100 Hz. It removes particularly well the line noise and other high-frequency noise [12], [23]. For online classification, signals were subsampled to 100 Hz without prior filtering. The continuous signals were then segmented into epochs between -150ms and 800 ms relative to each stimulus onset, using the first 150ms as a baseline.

Classification was based on spatio-temporal features [34] and preceded as in [12] and [16]. For each condition, the sample-wise *r^2^* coefficients (augmented with the sign of the difference) were calculated for *targets* vs. *nontargets*. Five time intervals in which those coefficients indicated highly discriminative information were generally determined heuristically [34] but sometimes manually adjusted by the experimenter (for example when the time intervals were chosen before the onset of the stimuli, the experimenter should choose one after the onset instead). Spatio-temporal features were determined from single-trials by averaging all samples within those intervals for each channel. This provides feature-vectors with dimensionality 63 (number of EEG channels) times 5 (number of time intervals), i.e. 315. For classification, regularized Linear Discriminant Analysis (LDA) with shrinkage regularization of the covariance matrix was used, see [34] for details. Classification was done for each stimulus, as a binary task to distinguish the *target* sub-trials from the *non-target* sub-trials. For online classification, in each selection period, one out of six stimuli had to be chosen (the one that was attended by the participant with highest probability). To that end, real-valued classifier outputs (distance to the separating hyperplane) were averaged for each of the six stimuli across all available repetitions and the stimulus with the highest scores was selected. In this study the number of repetitions was six for condition **AV** and ten for condition V*A.

In an offline analysis, the temporal distribution as well as the spatial distribution class discriminative information was investigated for each participant and condition. Therefore, the accuracy of a classifier was estimated which was trained either on all channels and sliding time intervals (window size  =  20 ms, step size  = 5 ms) or only one channel and heuristically determined time intervals.

As Schreuder et al. [38] has shown that the number of repetitions can significantly impact BCI performance, the BCI performance when using smaller numbers of repetitions was also estimated in offline analysis. Cross-validation was used to evaluate the classification performance based on the calibration data, also for the unimodal conditions with which no online spelling has been performed.

Though in general context the Information Transfer Rate (ITR) is a reasonable performance measure text, we consider the actual symbols per minute (SPM) for spelling applications is more straightforward and realistic [38]. For single level interfaces, a correct selection counts as +1 symbol. An erroneous selection counts as -1 symbol, to account for the added effort of performing a backspace. We used the formula described in [38] to investigate the SPM for each speller: 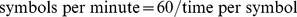
(1)

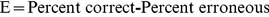
(2)

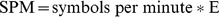
(3)


Need to notice that the *time per symbol* includes all the necessary overhead including countdown part, classification and feedback time.

## Results

In this study, we investigate and compare workload, ERP components, classification accuracy and spelling speed in each speller. We compare all of these features among condition **V**, **A**, and **AV** to investigate whether multi-sensory stimuli enhance BCI performance in the gaze-independent speller and to find the difference among brain response. We also present those results for condition V*A to study the possibility to use a BCI paradigm with two independent channels coding different information.

### Behavioral data

**Figure 3:**
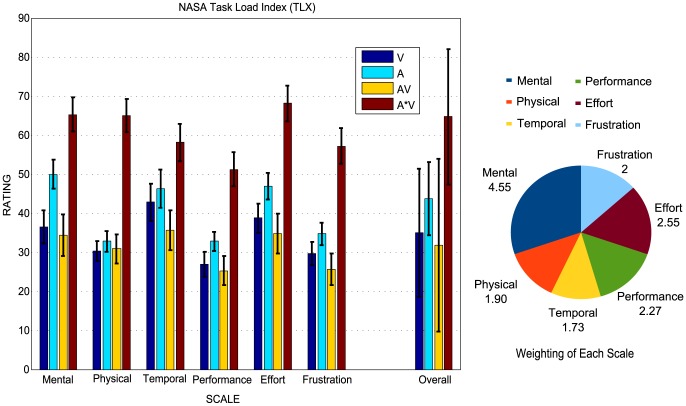
The NASA TLX workload and the overall weighted score of different conditions. The left column shows the mean rating with the standard deviation (SD) for each subscale and the overall weighted workload for each condition. The pie chart shows the grand-averaged weighting for each subscale. The total weighting is 15, due to 15 pair-wise comparisons of the sub-scales (see section 2.4).

The subjective workload that was imposed in each spelling condition was estimated based on the NASA TLX questionnaire. The left column of Figure 3 shows the mean rating with SD (standard deviation, stands for the standard deviation of the sampling distribution of the rating score) for each subscale and the overall weighted workload with SEM (standard error of the mean) for each condition. Also the grand averaged weights of the subscales were plotted on the right of the figure.

Results show that condition **AV** has the lowest, while condition V*A has the highest subjective workload for each sub-scale and overall. The data of workload has an approximate Gaussian distribution (K-S test, p>0.05), and they have equal sample variances (Levene tests, p> 0.05). Univariate ANOVA of the workload with factor *condition* and *subscale* was conducted. The results show significant effects of factors *condition* (p <.001) and *subscale* (p <.001), but no significant effects on condition*subscale (p  = .967). Pairwise comparison was also conducted on *conditions*. Significant differences were found for each comparison of conditions **A**, **AV**, and V*A (p <.050). Condition V and V*A also have significant difference (p <.001). The overall workload of all 4 conditions follow an approximate Gaussian distribution (K-S test, p  = .980).The results do not show significant difference among conditions **V**, **A**, and **AV** (p>.01), but significant difference (p <.01) between condition A*V and the other three conditions (**V, A, AV**). Moreover, the pie chart plot of weightings for the subscales reveals the subscale *Mental* (*30.3*%) to be the most important factor of workload, followed by *Effort* (*17.0*%).

### Event-related potentials

**Figure 4:**
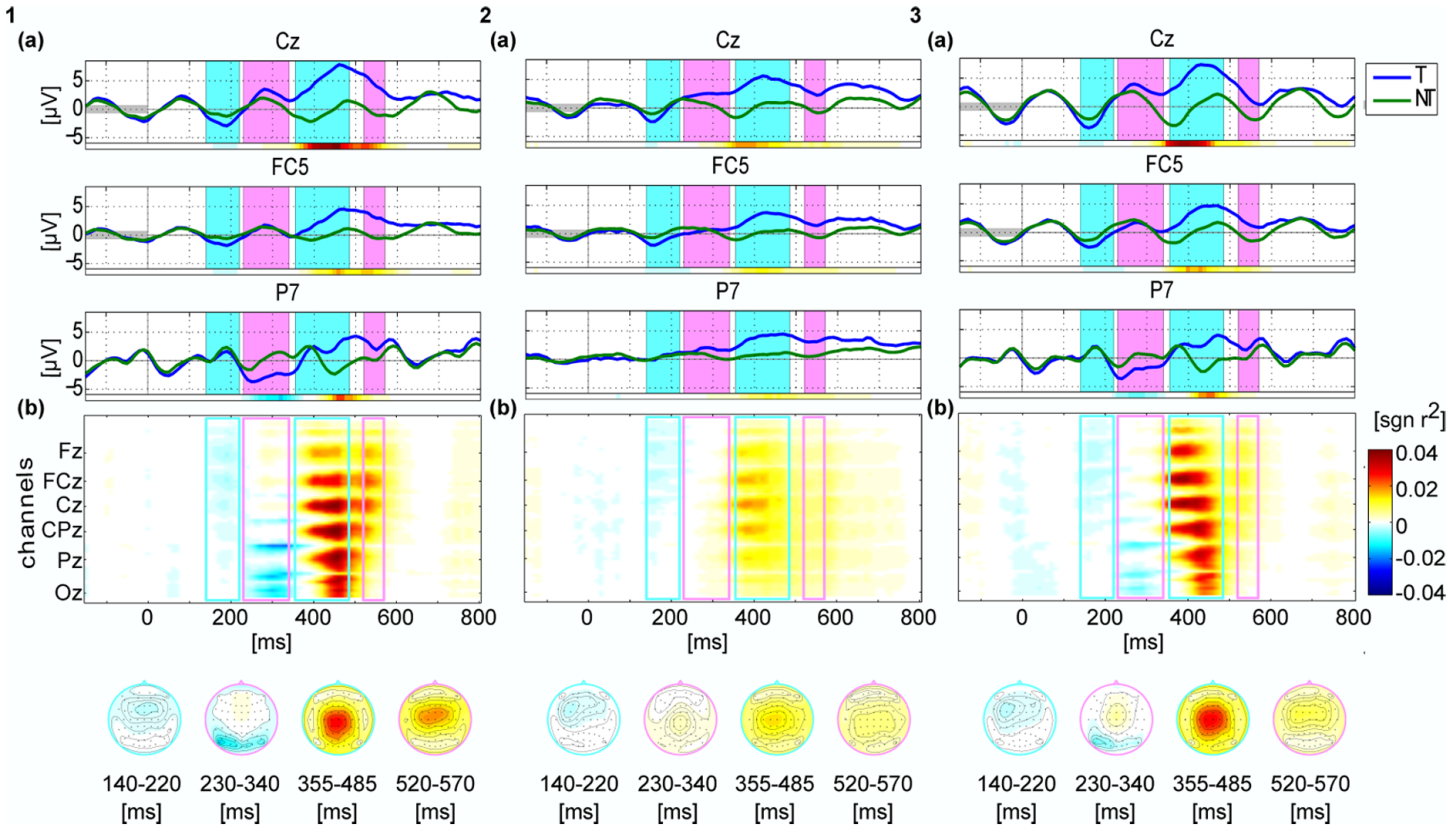
Grand averaged ERP and spatio-temporal diversity of the class-discriminative information for the first 3 conditions. Conditions are arranged in columns (left: **V**, middle: **A**, right: **AV**). All plots share the same color scale. The top row shows the ERPs for targets and non-targets of three selected electrodes Cz, FC5 and P7. The pink and green shade areas in each plot marked the time intervals, for which the scalp maps are shown at the bottom also colored accordingly. The colored bar underneath of each plot gives the signed correlation coefficient (sign *r^2^*). It indicated the difference between target and non-target classes for the chosen channel. In the middle, the spatio-temporal distribution of class-discriminative information was shown as a matrix plot, under which also the scalp maps of the chosen intervals were shown depicting the averaged *r^2^* values within the time intervals. The matrix plot shows the signed *r^2^* values for each EEG channel and for each time point. The light-blue and light-magenta rectangles depicted the chosen time interval as the shaded area in the top rows.

Figure 4 depicts the grand-average (N = 15 participants) of the event-related potentials (ERPs) and spatial-temporal diversities of the class-discriminative information of the first 3 conditions (left: **V**, middle: **A**, right: **AV**). FC5 electrode was used in [27] for the early negative auditory ERP components. In visual P3-speller literatures, PO7, P7 and (or) P8 were used to check the early negative ERP components. Also concerning the ERP scalp maps in Figure 4, we choose the three channels (Cz, FC5 and P7) to analysis. These three channels of grand averaged ERPs are plotted on the top of the figure. The matrix plots under the ERPs show the spatial-temporal discriminative information (signed *r^2^*) of *targets* vs. *non-targets* in each condition. To compare each component across different conditions, scalp maps of the class-discriminative information (*r^2^*) are shown for 4 fixed time intervals, which are marked in the ERP plots. The components shown in this 4 time intervals are denoted as 'N1', 'N2', 'P3' and 'P4', according to the polarity and the order of the components.

In condition **V**, there is a prominent negative component 250-350 ms after stimulus onset located at lateral parieto-occipital electrodes (P7, P9) with higher amplitudes on the left hemisphere. This component is referred to as N2. The ERPs at P7 in Figure 4.1a depicts the difference of grand average ERPs between *targets* and *nontargets*. As expected, a positive component is observed approximately 350-560 ms (channel Cz) after the stimulus onset (referred to as P3 components). As seen from the spatial-temporal distribution matrix plot (Figure 4.1b), the positive component starts from the centro-parietal area and extends to the surrounding electrodes. The decay of the discriminative information of this component progresses from occipital to frontal electrodes, see Figure 4.1b.

In condition **A**, all components have lower amplitudes compared to the corresponding components in condition **V**, which is in agreement with existing literature. An early negative component observed at approximately 150-230 ms (referred as N1 component) occurs at fronto-central areas. As already observed by Höhne et al. [27], this component is stronger on the left hemisphere than on the right hemisphere. The P3 component starts at around 360 ms with a focus in the central area.

Figure 4.3 depicts the ERPs and topographies of the class-discriminative information in condition **AV**. The N1 component in condition **AV** occurs around the frontal area with a higher amplitude on the left hemisphere, which is similar to condition **A**. The N2 component observed at parieto-occipital electrodes shows a similar but weaker response compared with that in condition **V**. The P3 component starts from the fronto-central area between 350 ms and 500 ms, showing an earlier discriminative response than that in condition **V** similar as in condition **A**. Surprisingly, the decay of the P3 component progresses from the front to the occipital area (Figure 4.3b) vary much in contract to the condition **V**.

**Figure 5:**
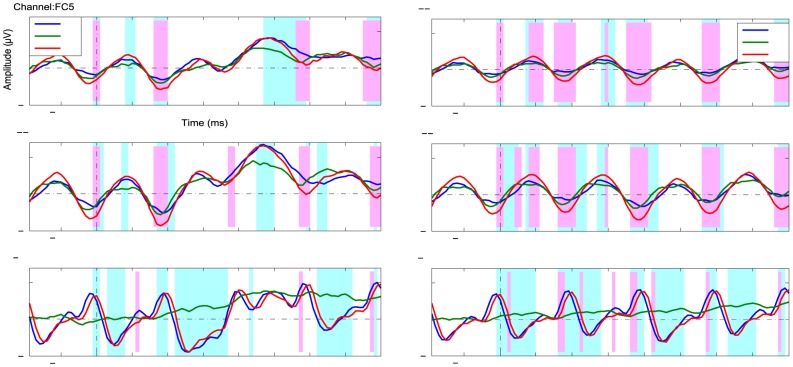
The ANOVA results of ERP response with factor *condition* (conditions V, A, and AV; left: Targets, right: Non-Targets). The time intervals with significant difference of ERP response for conditions **V**, **A**, and **AV** was marked light blue (p <.05). The pink-marked time-zones show the time intervals that have significant difference of conditions **V** and **AV** (p <.05).

Figure 5 shows the ANOVA (using Matlab) results of ERP response for conditions **V**, **A**, and **AV** (Left: Targets, right: Non-Targets). The ERP response has significant differences of 3 conditions were marked light blue (p <.05). Light pink marked time-zones show the significant difference between condition **V** and **AV** (p <.05). The early components of the ERP response after stimulus onset show significant differences both for Targets and Non-Targets, which might indicate that those difference was influenced by the stimuli properties of different stimuli. Light pinked zones could indicate the influence of auditory stimuli to visual stimuli response. No obvious significant difference was found for P300 components during 250–450 ms. Thus, the characteristics of the individual auditory stimuli influence rather the early ERP components, while later components are not affected.

**Figure 6:**
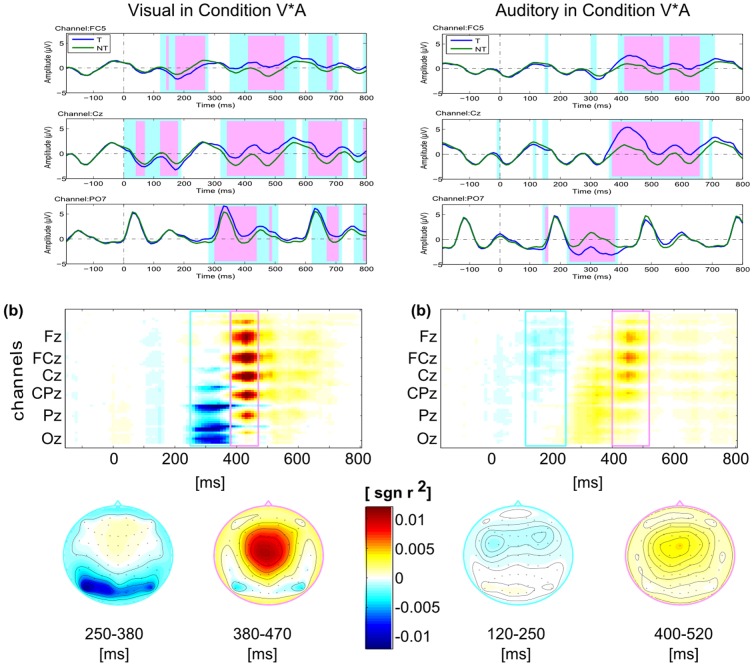
The ANOVA results of ERP response and class-discriminance (signed *r^2^*) maps of Targets versus Non-Targets in condition V*A. The two columns show the ERP responses for visual (left) and auditory (right) stimuli independently in condition V*A. The first row shows the ERP responses for the three selected electrodes FC5, Cz and PO7. The time intervals with significant difference of ERP response for Targets and Non-Targets was marked light blue (p <.05). The pink-marked time-zones show the time intervals that have more significant difference with p <.01. Spatio-temporal diversities of the class-discriminative information are shown in the second row. All plots share the same color scale (different scale in the colorbar compared to Figure 4). The spatial distribution of class-discriminant information is depicted with scalpmaps for two time intervals.

Figure 6 show the ERPs (as well as the ANOVA results) and class-discriminative information (signed *r^2^*) separately for visual and auditory stimuli in condition V*A (left: visual; right: auditory). The upper two subfigures show the ANOVA results of Targets versus Non-Targets for visual and auditory stimuli independently. Significant differences are found at large time zones for Target versus Non-Target ERP response. For the grand averaged visual response, the negative component at occipital area is observed at 250–380 ms after the visual stimulus onset, and the positive component at the central area occurs at 450 ms. The auditory negative component is observed at frontal areas from 100 to 250 ms and the positive response appears at the central area between 400 and 500 ms.

### Offline binary accuracy

**Figure 7:**
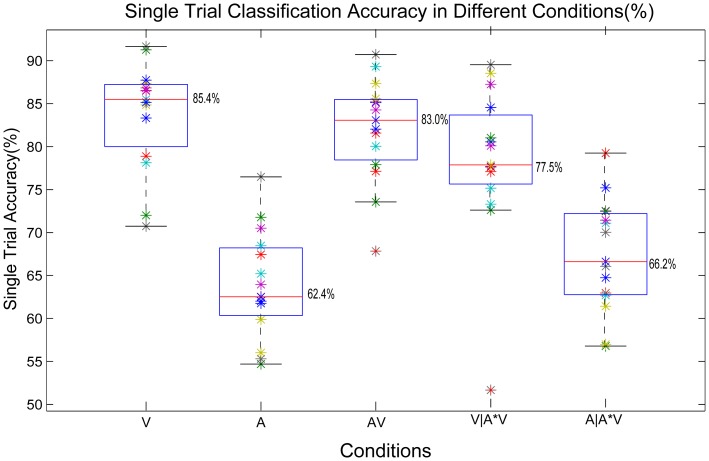
Single trial classification accuracies in different conditions for the binary *target* vs *non-target* discrimination. Accuracies are estimated by cross-validation on the calibration data using class-wise normalized loss function (chance level  =  0.5). Each colored '*' represents the accuracy for each participant in giving conditions. The edges of the blue box in each column reveal the 25% and 75% data range. The central red mark is the median accuracy overall the participants in the giving condition.

The single trial classification accuracies for each condition, which have an approximate Gaussian distribution (K-S test, p>0.05) and equal sample variances (Levene tests, p  = .875), were estimated and depicted in Figure 7. ANOVA of the accuracies with the factor condition was conducted. As expected, the classification accuracies in condition **V** and **AV** are significantly better than that in condition **A** (*p <.001*). However, there is no significant difference between condition **V** and condition **AV** (*p  =  1*). No significant differences were found between V and V|A*V as well as between A and A|A*V (p>.05).

**Figure 8:**
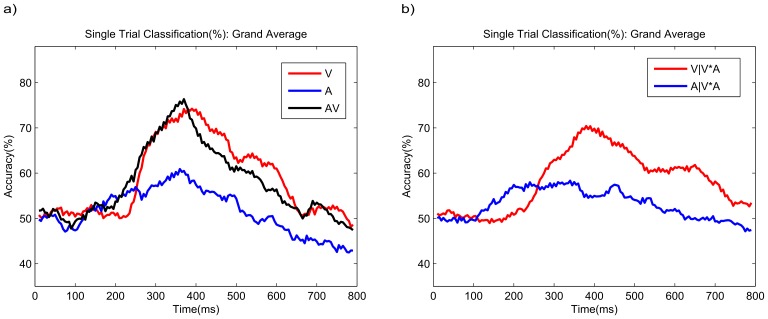
The grand averaged temporal distribution of discriminative information for each condition. a) The single trial classification for the unimodal spellers and Combined Speller (red: condition **V**; blue: condition **A**; black: condition **AV**). b) The temporal distribution of the single trial classification accuracy for condition V*A (red: visual classification; blue: auditory classification). The time window used in this study is 20 ms with a time step of 5 ms.

Figure 8.a depicts the grand average temporal distribution of discriminative information. To investigate which time intervals contribute most to classification success, a time window of 20 ms and time step of 5 ms were used to calculate the temporal distribution of the classifications. Cross-validation results of those features for each time point provide a temporal distribution of the multivariate discriminative information. The curve for condition **A** shows lower classification accuracy compared to condition **V** for the whole time period except for the early interval 200–260 ms. Condition **AV** effectively exploits the early components of both modalities with accuracies above both unimodal conditions between 200 and 300 ms. However, starting from about 380 ms the results of **AV** are inferior to those of **V**, which is in line with the observation of the earlier decay of the P3 component in the bimodal condition (see Figure 4).

Analogously to Figure 8.a, Figure 8.b shows the temporal evolution of the discriminative information for condition V*A. It is quite similar to the results for the unimodal conditions in Figure 8.a. However, surprisingly, for the auditory stimuli in the Parallel-Speller (V*A), the discriminative information starts earlier than in the unimodal condition **A**, which may be due to an increased level of attention.

**Figure 9:**
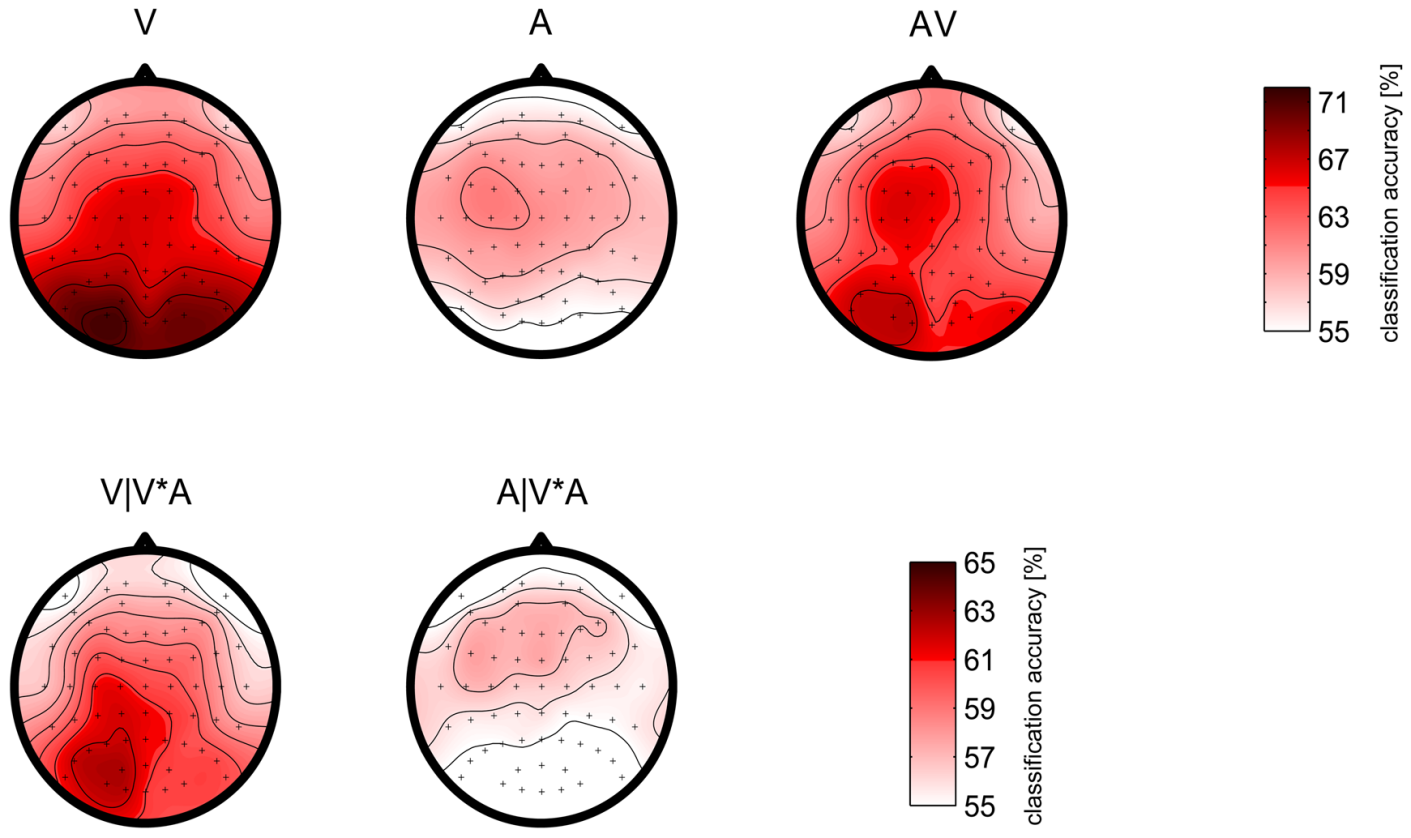
Spatial distribution of classification performance. Classification was made for each electrode individually. The time intervals were chosen by a heuristic between [0 800] after the stimulus onset. The grand average for each of the condition is shown and the binary classification accuracies are indicated by color gradients. The plots in the first row use the same color scale as shown on the right of that row. The figures in the second row use a different color bar as shown on the right of the second row.

Complementary to Figures 8, the spatial distribution of the discriminative information is displayed in Figure 9. The top row shows the results of the first 3 conditions (**V**, **A**, **AV**). In condition **V**, higher accuracies of the occipital electrodes suggest that visual and visual-attentional components are essential ingredients for classification. Discriminative information from cognitive processes as reflected in the P3 is spatially more wide spread and therefore not so prominent is this display of single channel analysis. Classification accuracies for condition **A** show the importance of fronto-central electrodes in particular on the left hemisphere, which is in line with the ERP results shown in Figure 4. The asymmetry also exists in condition **AV**.

The classification accuracies in condition V*A are lower than in the other conditions. Therefore, in the second row a different scale for the colormap is used for better display. Apart from the lower absolute value, the maps of classification accuracy look similar to the maps for the corresponding uni-modal conditions in the row above. The occipital area of the map for V|V*A seems to show a stronger lateralization to the left side than the maps of the visual-only condition.

### Online Spelling Results

**Figure 10:**
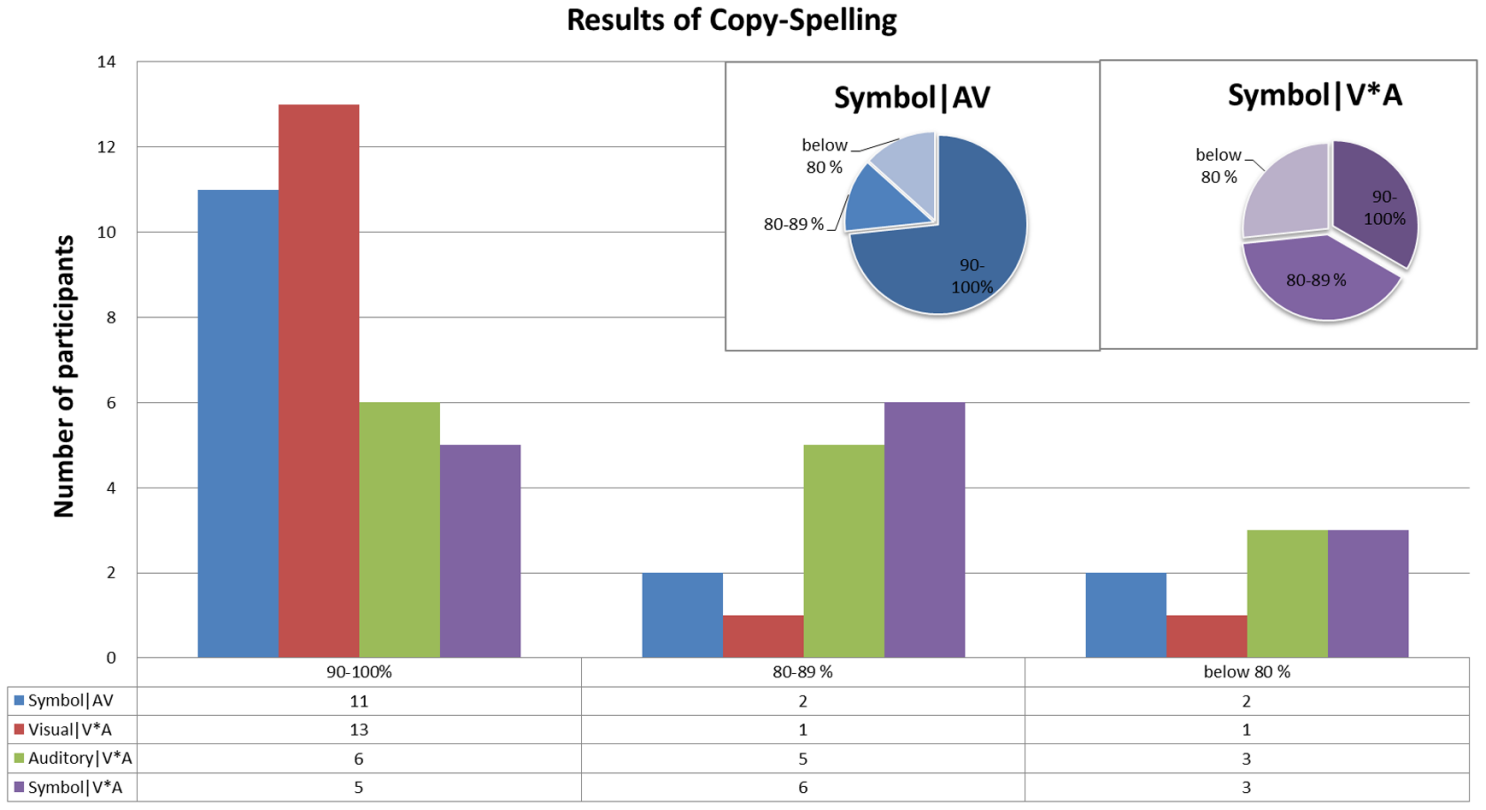
The distribution results of online copy spelling. The histogram shows the number of participants, whose classification accuracies were located in each accuracy scale. The accuracies were separated into 7 scales. ‘Symble|AV’ stands for the symbol selection accuracies in condition **AV**, and ‘Symbol|V*A’ stands for the symbol selection accuracies in condition V*A. The red bar, which is noted as ‘Visual|V*A’, represents the visual selection accuracies in condition V*A. The green bar, denoted as ‘Auditory|V*A’, shows the auditory selection accuracies distribution. The blue-dominant pie chart shows the proportion of each accuracy scale for the symbol selection accuracies in condition AV. The purple-dominant pie chart shows the proportion in condition V*A.

Figure 10 depicts the results of conditions **AV** and V*A in the online copy-spelling mode of this study. Each participant had to spell 20 symbols ('AUDIO_VISUAL_SPELLER') in both paradigms.

For condition **AV** (Combined-Speller), 6 repetitions of the stimuli were used for each selection, and two selection periods (group selection and symbol selection) have been conducted for each symbol. The blue bar in Figure 10 shows the symbol accuracies in condition **AV**. More than *73.3%* participants (11 in 15 participants) could get accuracy over *90%*. Only 2 of all the participants got accuracy under *80%.* The mean accuracy is *92.0%* (chance level <*3%*) with a speed of more than 2 symbols per minute.

In the Parallel-Speller (condition V*A) 10 repetitions of the stimuli have been used. The visual stimuli coded the group and the auditory stimuli coded the position of the symbol in the visual group. Since there is no pause or the need for correcting selection errors during copy-spelling, all of the participants finished this part within 10 min. The red and green bars in Figure 10 shows the visual (**V**) and auditory (**A**) selection accuracies separately. The visual selections for thirteen participants are over 90.0%, while only six participants could get accuracies over 90.0% for the auditory condition. Thus, the selection errors of the symbols are mostly due to an error of the auditory-based selection. Eleven of the participants achieved at least *80.0*% accuracy. Four participants spelled less than 16 correct symbols, thus displaying a selection accuracy of below 80%. All users could spell online with a mean accuracy of 87.7% (chance level <3%) showing a competitive average speed of 1.65 symbols per minute.

**Figure 11:**
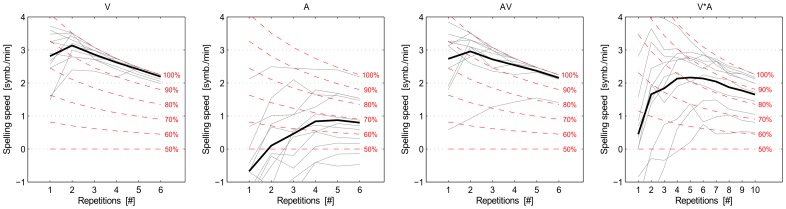
Spelling speed for each of the 4 conditions plotted against the number of repetitions. Thin gray lines depict results for single participants and the solid black line depicts the mean. Red dashed lines represent the spelling speed for fixed levels of symbol selection accuracy. Spelling accuracy for the empirical data (solid black line) can be deduced by comparing the black solid line to the red dashed lines. The accuracy is based on the calibration data for each condition.

We also performed offline simulations where we determined the spelling speed (symbols/minute) as a function of the number of the sequences using the calibration data. Figure 11 shows the results for different conditions. ANOVA measures with factors *condition* and *number of repetitions* was conducted to investigate the significance of each factor for the first 3 conditions. The results show significant effects of factors *condition* (*p <.001*), *number of repetitions* (*p <.001*) but no significant effect of the factor *condition* * *repetition* (*p  = .210*). However, concerning only the offline stimulation results of the spelling speed for conditions **V** and **AV**, there were significant effects of the *repetition* (*F  =  21.996, p  =  0*) factor, but no significant effect of the *condition* (*F  =  3.125, p  =  0.079*) and the *condition* * *repetition* (*F = .067, p  = .997*) factors.

## Discussion

To answer the questions mentioned in section 1, two ways of combining visual and auditory stimuli were investigated: the first one employed simultaneous visual and auditory stimuli which represent the same information (called Combined-Speller, denoted as AV). The second one used two independent streams of visual and auditory stimuli which allow integrating two selection periods into a single one (called Parallel-Speller, denoted as V*A). There were two central goals in this study: first, to evaluate the advantages and disadvantages of the Combined-Speller and to compare it with the uni-modal paradigms (comparing workload, ERP response and discrimination information); second, to demonstrate the feasibility and usability of the Parallel-Speller (discussing the ERP response, spelling speed and so on), in which two independent decisions are made parallel.

### Comparing the Combined-Speller with uni-modal paradigms

#### Workload

In our study, workload was used as a measure of usability of a system. Mental demand is the major factor for the weighted overall workload. Thus it is important to reduce the mental workload when operating a BCI system. It was reported in [36] that the workload for an auditory speller was significant higher than that for a (gaze-dependent) visual speller. Similar result but not significant difference was obtained here for an auditory compared with a gaze-independent visual paradigm. In particular, participants mentioned the difficulty of ignoring the non-targets, which had the same syllables or came from the same speakers as the target stimuli. However, when being asked after the whole experiment, some participants felt it was easier to attend uni-modal auditory speller compared with uni-modal visual speller, especially after having to focus on the center of the screen for such long time. The workload for the Combined-Speller (condition AV) was insignificantly lower than that for the mono-visual and mono-auditory paradigms. However, Participants (except one) mentioned that they were more relaxed during the Combined-Speller without always intensively focusing to either the visual stimuli or the auditory stimuli. Some participants regarded the target auditory stimuli as cues for the coming visual target, while others regarded the visual stimuli as cues instead. Thus, one can get to the conclusion that with an increasing runtime of the experiment, the Combined-Speller is the best choice with respect to workload, not only because the low workload it needs during spelling, but also the changing of modality to release the mental workload.

#### The ERP and class-discrimination information

Belitski et al. [30] reported that multi-modal (audio+visual) stimulation increased the average strength of the stimulus response in matrix speller style BCIs, when compared to either visual or auditory stimulation in isolation. Thurling et al. [31], however, reported an enhanced N1 and reduced P300 in bimodal visual-tactile paradigm.

Detailed results were derived in our study. From Figure 4, we could find that the peak of the visual N1 component of Combined-Speller is shifted to the left hemisphere. This might be due to the evoked responses of the auditory stimulation. The discriminability of the N2 component is reduced compared to the uni-modal visual speller, with a possible explanation that the positive response caused by the auditory stimuli affected the negative response for visual stimuli at the same time. Focusing on the following positive components, we find that the P3 component in the Combined-Speller had a shorter latency than in the mono-visual paradigm. While the P3 component in the unimodal visual speller featured a second component with more frontal focus, this sub-component was absent in the bimodal condition. However, the exact mechanism of the response in the Combined-Speller remains to be investigated in further studies.

From the statistical analysis of the ERPs (Figure 5) for 3 channels, we could find that the early ERP components show significant improvements (i.e. increased discriminability) in the Combined-Speller compared to the uni-modal visual and auditory spellers. The time intervals marked in light blue and pink show the significant difference amongst the three conditions. Thus, the bimodal stimulation in condition **AV** mainly impacts the early ERP components such as P1 and N1. No obvious significant difference were found during P300 component from 250 – 450 ms between conditions **V** and **AV**. However, both conditions yield to significantly higher P300 amplitude than condition A. Since channel PO7 reflects mostly on the visual processing, conditions **V** and **AV** don't show significant differences at that channel.

The spatial information of the discriminant information shown in Figure 9 reveals two important findings: the visual response area was affected by the auditory stimuli, with the central higher classification area shifting left as the auditory stimuli; the occipital electrodes featured less classification accuracies in Combined-Speller than that in the uni-modal visual speller. As possible reason, it can be speculated that less attention was allocated to the visual stimuli due to the concurrent auditory stimulation.

Thus, visual stimuli with concurrent auditory stimuli have significant difference between single modal visual and auditory stimuli. ERP difference was mostly on early exogenous components during 0 – 200 ms after stimuli onset and in frontal and central electrodes.

### Parallel-Speller

In previous studies [21], [30], [31], multi-modal spelling paradigms all used visual and auditory stimuli to convey the same information. Our study used for the first time visual and auditory stimuli to convey different information in parallel. The workload of the parallel speller is considerably higher than for the other three spellers.

Figure 6 provides an overview over the class-discriminative information for the Parallel-Speller. The negative and positive components occurred at specific area were clearly found for visual and auditory stimuli respectively in Parallel-Speller. It shows that the visual and auditory stimuli could work independently, though having a higher workload used (according to Figure 3).

Besides, the temporal and spatial distribution of single trial classification accuracy also showed a possible classifier for visual and auditory stimuli respectively, similar though lower accuracies and tightened response area as uni-modal visual and auditory stimuli.

### Performances of Combined-Speller and Parallel-Speller

Riccio et al. [17] provides a comparison of most BCI spellers with visual and (or) auditory stimuli. The comparison was discussed and depicted in a table (Table 1 in [17]) through the accuracy, bits per symbol, symbols per minute and bits per minute of the BCIs. Most spellers using auditory or visual + auditory stimuli have accuracies less than 70% and ITR less than 1.9 bits per minute if the total number of selections is more than twenty. Concerning the visual stimuli, only one (out of thirteen) speller has an ITR up to 10 bits per minute. We describe the Combined-Speller and the Parallel-Speller. Compared to the gaze-independent spellers reviewed by Riccio et al. [17], our paradigms featured competitive selection accuracies (94.9% for Combined-Speller and 85.9% for Parallel-Speller) and overall BCI performances (ITR of 10.01 and 8.85 bits per minute respectively).

The spelling speed (symbols per minute), which is one of the evaluation of the feasibility of spelling system, shows that for the Parallel-Speller most participants could spell 2 symbols per minute when 5 repetitions were used. Two participants could even spell about 3.5 symbols per minute when 2 repetitions were used, which is comparable even better than other paradigms. All of the above results prove the feasibility and usability of the Parallel-Speller.

Furthermore, slower spelling speeds have been obtained with the parallel speller. Apart from the slower spelling speed, several participants indicated a preference for the Parallel-Speller, due to the fact that there is only one period to select a symbol. Several measures to be taken in future developments can be expected to give improvement in this respect: (a) Due to the more difficult task, some training might be required. (b) Given the difference in detectability in the sensory domains, an uneven distribution of the auditory and visual stimuli might be beneficial. For example, if symbols are distributed in eight groups of four each, double the number of repetitions for auditory stimuli can be collected. (c) The current study used a considerable 'overhead' for the selection procedure in the Parallel-Speller. The ‘overhead’ contains the countdown part (hint for the target stimuli or a blank period for remember the targets, and the pre-flashing of the last digits), the classification time (to compute the result selections) and feedback (to show the selected group or symbols on the screen), more information was described in section 2.3. In this study, the stimulation time for each symbol in Parallel-Speller was about 18 s, while the 'overhead' (the countdown, classification and feedback) occupied about 10 s. For Combined-Speller, the stimulation time for each symbol was 2*7.2 s, while the 'overhead' was about 2*6.1 s. We assume that after practicing with the Parallel-Speller, the assistant time could reduce to 6.1 s as in Combined-Speller. (d) An optimal stopping method (Schreuder et al, 2013) could be employed to enhance the performance for the Parallel-Speller.

The Parallel-Speller is a novel approach for an ERP speller, which provides new insight into multisensory processing. Moreover, it represents a way to practice these two human sensory channels.

## Conclusions

In a multisensory world, it might be advisable to also use multisensory stimulation for BCI applications. We approached this topic by comparing unimodal stimuli (from either visual or auditory domain), with multisensory stimuli from both domains. We studies two kinds of multisensory integration in an ERP-based BCI speller: the Combined-Speller and the Parallel-Speller.

For the Combined-Speller, most participants pointed out the positive aspect that it is not necessary to continuously locate attention to a fixed modality. The ERP response as well as the distribution of discriminative information was observed to be different for combined-speller compared to unimodal stimuli. It remains an area of future research to exploit such differences. Comparing the Combined-Speller to uni-modal paradigms we found shorter latencies, lower amplitudes, as well as a shift of the temporal and spatial distribution of discriminative information. As it was already suggested in this study, the Combined-Speller is a good choice for BCI speller with a low workload.

Moreover, a novel multimodal stimulus paradigm, called ‘Parallel-Speller’, was introduced. The Parallel-Speller combines two independent streams of stimuli, enabling a 1-out-of-36 decision with a single step. However, the workload is increased compared to all other conditions. The results for its classification and brain response showed that it is possible to apply such a truly multimodal paradigm. We hope that the new way of combining sensory modalities could stimulate further discussions and novel applications.

## References

[1] Wolpaw JR, Wolpaw EW (2012) Brain-Computer Interfaces: Principles and Practice. Oxford University Press.

[2] Dornhege G (2007) Toward Brain-Computer Interfacing. MIT Press.

[3] Wolpaw JR, McFarland DJ (2004) Control of a two-dimensional movement signal by a noninvasive brain-computer interface in humans. Proc Natl Acad Sci USA 101: 17849–54.. 15585584 10.1073/pnas.0403504101 PMC535103

[4] Blankertz B, Dornhege G, Krauledat M, Müller KR, Curio G (2007) The non-invasive Berlin brain-computer interface: fast acquisition of effective performance in untrained subjects. Neuroimage 37: 539–50.. 17475513 10.1016/j.neuroimage.2007.01.051

[5] Mak JN, Wolpaw JR (2009) Clinical applications of brain-computer interface: current state and future prospects. IEEE Rev Biomed Eng 2: 187–99.. 20442804 10.1109/RBME.2009.2035356 PMC2862632

[6] Farwell LA, Donchin E (1988) Talking off the top of your head: toward a mental prosthesis utilizing event-related brain potentials. Electroencephalogr Clin Neurophysiol 70: 510–23.. 2461285 10.1016/0013-4694(88)90149-6

[7] Zhang D, Song H, Xu H, Wu W, Gao S, et al (2012) An N200 speller integration the spatial profile for the detection of the non-control state. J.Neural Eng 9: 026016.. 22414615 10.1088/1741-2560/9/2/026016

[8] Bin G, Gao X, Wang Y, Li Y, Hong B, et al (2011) A high-speed BCI based on code modulation VEP. J Neural Eng 8: 025015.. 21436527 10.1088/1741-2560/8/2/025015

[9] Sellers EW, Vauqhan TM, Wolpaw JR (2010) A brain-computer interface for long-term independent home use. Amyotroph Lateral Scler 11 (5): 449–55.. 10.3109/17482961003777470 20583947

[10] Brunner P, Joshi S, Briskin S, Wolpaw JR, Bischof H, et al (2010) Does the 'P300' speller depend on eye gaze?. J Neural Eng 7: 056013.. 20858924 10.1088/1741-2560/7/5/056013 PMC2992970

[11] Treder MS, Blankertz B (2010) (C)overt attention and visual speller design in an ERP-based brain-computer interface. Behavioral and Brain Function 6: 28.. 10.1186/1744-9081-6-28 PMC2904265 20509913

[12] Treder MS, Schmidt NM, Blankertz B (2011) Gaze-independent brain–computer interfaces based on covert attention and feature attention. J Neural Eng 8: 066003.. 21975312 10.1088/1741-2560/8/6/066003

[13] Schaeff S, Treder MS, Venthur B, Blankertz B (2012) Exploring motion VEPs for gaze-independent communication. J Neural Eng 9: 045006.. 22832017 10.1088/1741-2560/9/4/045006

[14] Liu Y, Zhou Z, Hu D (2011) Gaze-independent brain-computer speller with covert visual search tasks. Clin Neurophysiol 122(6): 1127–36.. 21163695 10.1016/j.clinph.2010.10.049

[15] Aloise F, Aricò P, Schettini F, Riccio A, Salinari S, et al (2012) A covert attention P300-based brain-computer interface: Geospell. Ergonomics 55(5): 538–551. 22455372 10.1080/00140139.2012.661084

[16] Acqualaqna L, Blankertz B (2013) Gaze-independent BCI-spelling using rapid serial visual presentation (RSVP). Clin Neurophysiol 124(5): 901–8.. 23466266 10.1016/j.clinph.2012.12.050

[17] Riccio A, Mattia D, Simione L, Olivetti M, Cincotti F (2012) Eye-gaze independent EEG-based brain-computer interfaces for communication. J Neural Eng 9(4): 045001.. 22831893 10.1088/1741-2560/9/4/045001

[18] Brouwer A-M, van Erp JBF, Aloise F, Cincotti F (2010) Tactile, Visual, and Bimodal P300s: Could Bimodal P300s Boost BCI Performance? SRX Neuroscience 2010: 1–9..

[19] Van der Waal M, Severens M, Geuze J, Desain P (2012) Introducing the tactile speller: an ERP-based brain–computer interface for communication. Journal of Neural Engineering 9: 045002.. 22831906 10.1088/1741-2560/9/4/045002

[20] Kübler A, Furdea A, Halder S, Hammer EM, Nijboer F, et al (2009) A brain-computer interface controlled auditory event-related potential (p300) spelling system for locked-in patients. Annals of the New York Academy of Sciences 1157: 90–100.. 19351359 10.1111/j.1749-6632.2008.04122.x

[21] Klobassa DS, Vaughan TM, Brunner P, Schwartz NE, Wolpaw JR, et al (2009) Toward a high-through put auditory P300-based brain-computer interface. Clin Neurophysiol 120(7): 1252–61.. 19574091 10.1016/j.clinph.2009.04.019 PMC2729552

[22] Halder S, Hammer EM, Kleih SC, Bogdan M, Rosenstiel W, et al (2013) Prediction of auditory and visual p300 brain-computer interface aptitude. PloS one 8: e53513.. 23457444 10.1371/journal.pone.0053513 PMC3573031

[23] Schreuder M, Blankertz B, Tangermann M (2010) A New Auditory Multi-Class Brain-Computer Interface Paradigm: Spatial Hearing as an Informative Cue. PloS One 5: e9813.. 20368976 10.1371/journal.pone.0009813 PMC2848564

[24] Schreuder M, Rost T, Tangermann M (2011) Listen, you are writing! Speeding up online spelling with a dynamic auditory BCI. Front Neuroscience 5: 112.. 10.3389/fnins.2011.00112 PMC3192990 22016719

[25] Höhne J, Schreuder M, Blankertz B, Tangermann M (2011) A Novel 9-Class Auditory ERP Paradigm Driving a Predictive Text Entry System. Front Neurosci 5: 99. 21909321 10.3389/fnins.2011.00099 PMC3163907

[26] Höhne J, Tangermann M (2014) Towards user-friendly spelling with an auditory brain-computer interface: the CharStreamer paradigm. PloS One 9: e98322.. 24886978 10.1371/journal.pone.0098322 PMC4041754

[27] Höhne J, Krenzlin K, Dähne S, Tangermann M (2012) Natural stimuli improve auditory BCIs with respect to ergonomics and performance. J Neural Eng 9: 045003.. 22831919 10.1088/1741-2560/9/4/045003

[28] Teder-Sälejärvi WA, Di Russo F, McDonald JJ, Hillyard SA (2005) Effects of spatial congruity on audio-visual multimodal integration. Journal of Cognitive Neuroscience 17: 1396–409 doi:10.1162/0898929054985383 16197693 10.1162/0898929054985383

[29] Campanella S, Bruyer R, Froidbise S, Rossignol M, Joassin F, et al (2010) Is two better than one? A cross-modal oddball paradigm reveals greater sensitivity of the P300 to emotional face-voice associations. Clincal Neurophysiology 121: 1855–1862.. 10.1016/j.clinph.2010.04.004 20434394

[30] Belitski A, Farquhar J, Desain P (2011) P300 audio-visual speller. J Neural Eng 8: 025022.. 21436523 10.1088/1741-2560/8/2/025022

[31] Thurling ME, Brouwer Am, Van Erp JBF, Blankertz B, Werkhoven PJ (2012) Does bimodal stimulus presentation increase ERP components usable in BCIs? J Neural Eng 9: 045005.. 22831989 10.1088/1741-2560/9/4/045005

[32] Talsma D, Senkowski D, Soto-Faraco S, Woldorff MG (2010) The multifaceted interplay between attention and multisensory integration. Trends Cogn Sci 14: 400–10. 20675182 10.1016/j.tics.2010.06.008 PMC3306770

[33] Senkowski D, Talsma D, Grigutsch M (2007) Good times for multisensory integration: effects of the precision of temporal synchrony as revealed by gamma-band oscillations. Neuropsychologia 45: 561–571.. 16542688 10.1016/j.neuropsychologia.2006.01.013

[34] Blankertz B, Lemm S, Treder MS, Haufe S, Müller KR (2011) Single-trial analysis and classification of ERP components-a tutorial. Neuroimage 56: 814–825. 20600976 10.1016/j.neuroimage.2010.06.048

[35] Pasqualotto E, Simonetta A, Gnisci V, Federici S, Olivetti M (2011) BelardinelliToward a usability evaluation of BCIs. Int J Bioelectromagn 13: 121–22..

[36] Riccio A, Leotta F, Bianchi L, Aloise F, Zickler C, et al (2011) Workload measurement in a communication application operated through a P300-based brain-computer interface. J Neural Eng 8: 025028.. 21436511 10.1088/1741-2560/8/2/025028

[37] Käthner I, Ruf CA, Pasqualotto E, Braun C, Birbaumer N, et al (2013) A po auditory P300 brain-computer interface with directional cues. Clin Neurophyisol 124: 327–38.. 10.1016/j.clinph.2012.08.006 22959257

[38] Schreuder M, Höhne J, Blankertz B, Haufe S, Dickhaus T, et al (2013) Optimizing event-related potentail based brain-computer interface: a systematic evaluation of dynamic stopping methods. J Neural Eng 10: 036025.. 23685458 10.1088/1741-2560/10/3/036025

