# Correction: Exploring Combinations of Auditory and Visual Stimuli for Gaze-Independent Brain-Computer Interfaces

**DOI:** 10.1371/journal.pone.0157284

**Published:** 2016-06-03

**Authors:** The PLOS ONE Staff

The axes of Fig 5 are incorrectly printed. The authors have provided a corrected version here. The publisher apologizes for the error.

**Fig 5:**
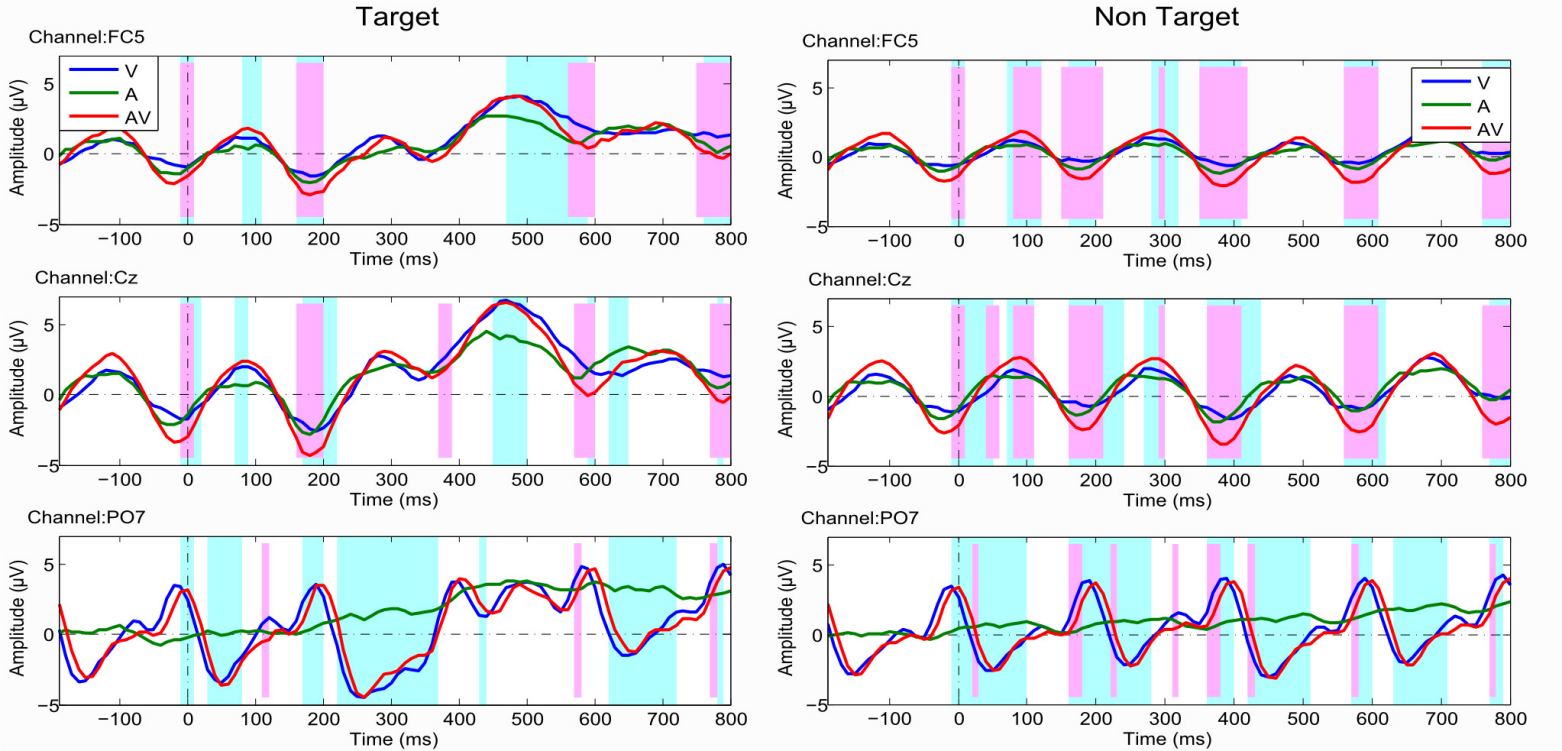
The ANOVA results of ERP response with factor condition (conditions V, A, and AV; left: Targets, right: Non-Targets). The time intervals with significant difference of ERP response for conditions V, A, and AV was marked light blue (p < .05). The pink-marked time-zones show the time intervals that have significant difference of conditions V and AV (p < .05).
